# Supplementary material for: Structural characterization of the oligomerization of full-length Hantaan virus polymerase into symmetric dimers and hexamers
Source: Nat Commun. 2024 Mar 13;15:2256. doi: 10.1038/s41467-024-46601-4 (PMC10937945; doi:10.1038/s41467-024-46601-4)
Supplement: Supplementary file 1 — Supplementary Information [file 41467_2024_46601_MOESM1_ESM.pdf]

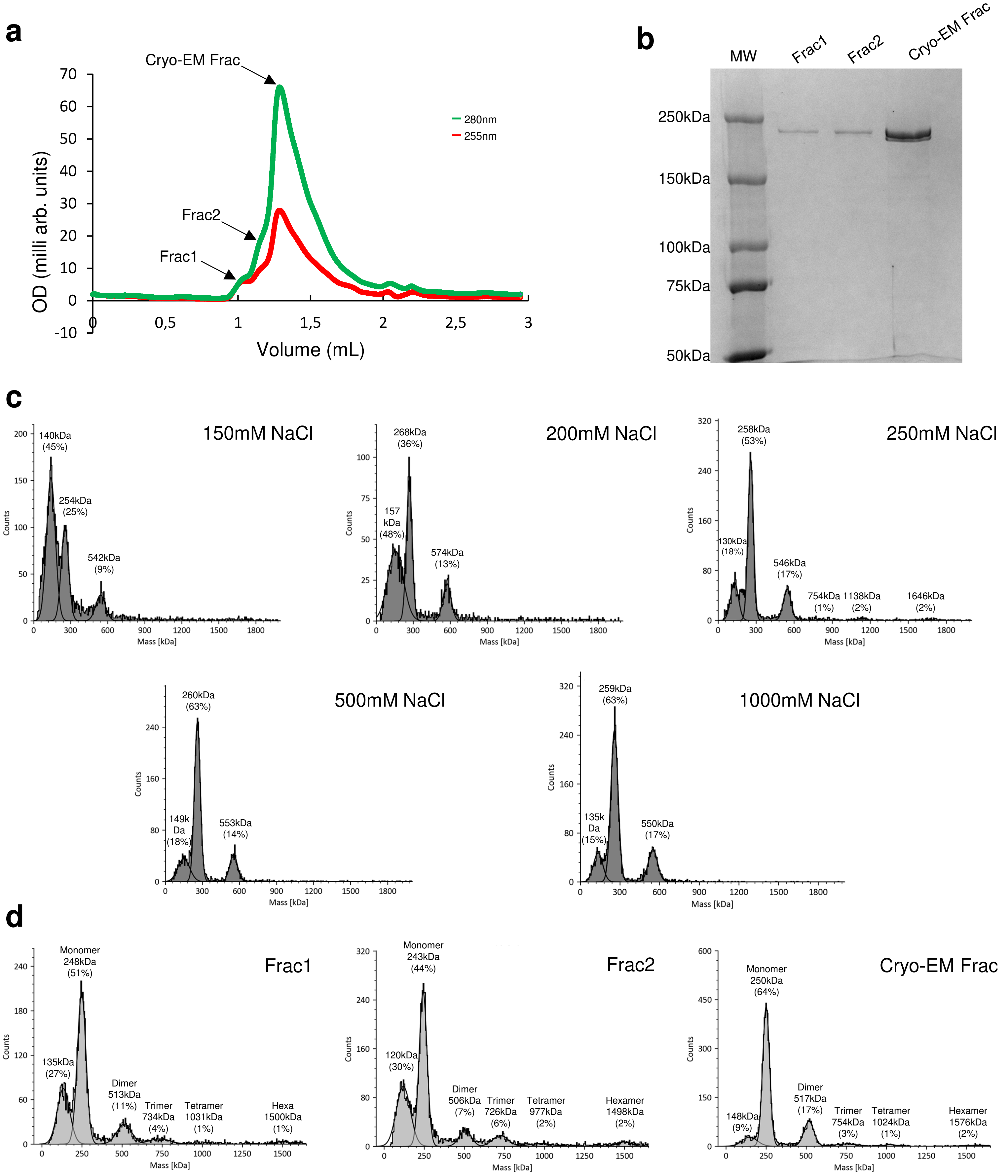

Supplementary Figure 1 HTNV-L purification and characterization of its oligomerization

a Size-exclusion chromatography profile of HTNV-L purification on a superdex 200 increase 3.2/300 column. The optical densities (OD) at 255 nm and 280 nm are respectively indicated in red and green as a function of the elution volume. The three fractions analyzed in **b** and **c** are indicated with arrows.

b SDS-PAGE gels of the 3 fractions indicated in **a**. The molecular weight marker (MW) is shown on the left. Source data are provided as a Source Data file.

c Mass photometry in buffers containing 30 mM HEPES, 5 mM TCEP and a concentration of NaCl that varies between 150 mM and 1M as indicated. The molecular mass in kDa and the percentage of each species are indicated.

d Mass photometry of the 3 fractions indicated in **a**. The mass in kDa and the percentage of each species detected are indicated.

a

protein name	mol. weight (Da)	coverage (%)	Band 150 kDa				Band 250 kDa			
			Pep	SC	iBAQ	Rank	Pep	SC	iBAQ	Rank
HTNV-LD97A	249123	75.75	184	250	2.60E+07	1	192	497	1.47E+08	1
LOW QUALITY PROTEIN: ubiquitin-40S ribosomal protein S27a-like	17887	24.52					3	3	2.85E+05	2
La-related protein 1-like	141647	38.85	46	62	5.27E+06	2	18	21	2.77E+05	3
Uncharacterized protein LOC113500194	131968	0.61					1	1	5.13+04	4
coproporphyrinogen oxidase	45966	1.78					1	1	3.32+04	5
Protein lingerer-like	100231	12.78	11	13	9.67E+05	3				
Cytochrome b	44198	1.83	1	1	3.89E+05	4				
Zinc finger CCHC domain-containing protein 8 homolog	80032	17.93	11	13	2.69E+05	5				

b

Band 150kDa

MGHHHHHHDYDIPTTENLYFQGMDKYREIHNKLKEFSPGTLTAVECIDYLDRLYAVRHDIVDQMIKHDWSDNKDSEEAIGKVLLFAGVPSNIITALEKKIIPNHPTGKSLKAFFK
MTPDNYKISGTTIEFVEVTVTADVDKGIREKKLKYEAGLTYIEQELHKFFLKGEIPQPYKITFNVVAVRTDGSNITTQWPSRRNDGVVQYMRLVQAEISYVREHLIKTEERAALE
AMFNLKFNISTHKSQPYYIPDYKGMEPIGANIEDLVDYSKDWLSRARNFSFFEVKGTAVFECFNSNEANHCQRYPMSRKPRNFLLIQCSLITSYKPATTLSDQIDSRRACSYILN
LIPDTPASYLIHDMAYRYINLTREDMINYYAPRIQFKQTQNVREPGTFKLTSSMLRAESKAMLDLLNNHKSGEKHGAQIESLNIASHIVQSESVSLITKILSDLELNITEPSTQE
YSTTKHTYVDTVLDKFFQNETQKYLIDVLKKTTAWHIGHLIRDITESLIAHSGLKRSKYWSLHSYNNGNVILFILPSKSLEVAGSFIRFITVFRIGPGLVDKDNLDTILIDGDSQ
WGVSKVMSIDLNRLLALNIAFEKALIATATWFQYYTEDQGQFPLQYAIRSVFANHFLLAICQKMKLCAIFDNLRYLIPAVTSLYSGFPSLIEKLFERPFKSSLEVYIYYNIKSLL
VALAQNNKARFYSKVKLLGLTVDQSTVGASGVYPSFMSRIVYKHYRSLISEVTTCFFLFEKGLHGNMNEEAKIHLETVEWALKFREKEEKYGESLVENGYMMWELRANAELAEQQ
LYCQDAIELAAIELNKVLATKSSVVANSILSKNWEEPYFSQTRNISLKGMSGQVQEDGHLSSSVTIIEAIRYLSNSRHNPSLLKLYEETREQKAMARIVRKYQRTEADRGFFITT
LPTRCRLEIIEDYYDAIAKNISEEYISYGGEKKILAIQGALEKALRWASGESFIELSNHKFIRMKRKLMYVSADATKWSPGDNSAKFRRFTSMLHNGLPNNKLKNCVIDALKQVY
KTDFFMSRKLRNYIDSMESLDPHIKQFLDFFPDGHHGEVKGNWLQGNLNKCSSLFGVAMSLLFKQVWTNLFPELDCFFEFAHHSDDALFIYGYLEPVDDGTDWFLFVSQQIQAGH
LHWFSVNTEMWKSMFNLHEHILLLGSIKISPKKTTVSPTNAEFLSTFFEGCAVSIPFVKILLGSLSDLPGLGYFDDLAAAQSRCVKALDLGASPQVAQLAVALCTSKVERLYGTA
PGMVNHPAAYLQVKHTDTPIPLGGNGAMSIMELATAGIGMSDKNLLKRALLGYSHKRQKSMLYILGLFKFLMKLSDETFQHERLGQFSFIGKVQWKIFTPKSEFEFADMYTSKFL
ELWSSQHVTYDYIIPKGRDNLLIYLVRKLNDPSIVTAMTMQSPLQLRFRMQAKQHMKVCRLDGEWVTFREVLAAANSFAENYSATSQDMDLFQTLTSCTFSKEYAWKDFLNGIHC
DVIPTKQVQRAKVARTFTVREKDQIIQNSIPAVIGYKFAVTVEEMSDVLDTAKFPDSLSVDLKTMKDGVYRELGLDISLPDVMKRIAPMLYKSSKSRVVIVQGNVEGTAEAICRY
WLKSMSLVKTIRVKPHKEVLQAVSIFNRKEDIGQQKDLAALKLCIEVWRWCKANSAPYRDWFQALWFEDKTFSEWLDRFCRVGVPPIDPEIQCAALMIADIKGDYSVLQLQANRR
AYSGKQYDAYCVQTYNEVTKLYEGDLRVTFNFGLDCARLEIFWDKKAYILETSITQKHVLKIMMDEVSKELIKCGMRFNTEQVQGVRHMVLFKTESGFEWGKPNIPCIVYKNCVL
RTSLRTTQAINHKFMITIKDDGLRAIAQHDEDSPRFLLAHAFHTIRDIRYQAVDAVSNVWFIHKGVKLYLNPIISSGLLENFMKNLPAAIPPAAYSLIMNRAKISVDLFMFNDLL
KLINPRNTLDLSGLETTGDEFSTVSSMSSRLWSEEMSLVDDDEELDDEFTIDLQDVDFENIDIEADIEHFLQDESSYTGDLLISTEETESKKMRGIVKILEPVRLIKSWVSRGLS
IEKVYSPVNIILMSRYISKTFNLSTKQVSLLDPYDLTELESIVRGWGECVIDQFESLDREAQNMVVNKGICPEDVIPDSLFSFRHTMVLLRRLFPQDSISSFY

Coverage: 58.44%

Band 250kDa

MGHHHHHHDYDIPTTENLYFQGMDKYREIHNKLKEFSPGTLTAVECIDYLDRLYAVRHDIVDQMIKHDWSDNKDSEEAIGKVLLFAGVPSNIITALEKKIIPNHPTGKSLKAFFK
MTPDNYKISGTTIEFVEVTVTADVDKGIREKKLKYEAGLTYIEQELHKFFLKGEIPQPYKITFNVVAVRTDGSNITTQWPSRRNDGVVQYMRLVQAEISYVREHLIKTEERAALE
AMFNLKFNISTHKSQPYYIPDYKGMEPIGANIEDLVDYSKDWLSRARNFSFFEVKGTAVFECFNSNEANHCQRYPMSRKPRNFLLIQCSLITSYKPATTLSDQIDSRRACSYILN
LIPDTPASYLIHDMAYRYINLTREDMINYYAPRIQFKQTQNVREPGTFKLTSSMLRAESKAMLDLLNNHKSGEKHGAQIESLNIASHIVQSESVSLITKILSDLELNITEPSTQE
YSTTKHTYVDTVLDKFFQNETQKYLIDVLKKTTAWHIGHLIRDITESLIAHSGLKRSKYWSLHSYNNGNVILFILPSKSLEVAGSFIRFITVFRIGPGLVDKDNLDTILIDGDSQ
WGVSKVMSIDLNRLLALNIAFEKALIATATWFQYYTEDQGQFPLQYAIRSVFANHFLLAICQKMKLCAIFDNLRYLIPAVTSLYSGFPSLIEKLFERPFKSSLEVYIYYNIKSLL
VALAQNNKARFYSKVKLLGLTVDQSTVGASGVYPSFMSRIVYKHYRSLISEVTTCFFLFEKGLHGNMNEEAKIHLETVEWALKFREKEEKYGESLVENGYMMWELRANAELAEQQ
LYCQDAIELAAIELNKVLATKSSVVANSILSKNWEEPYFSQTRNISLKGMSGQVQEDGHLSSSVTIIEAIRYLSNSRHNPSLLKLYEETREQKAMARIVRKYQRTEADRGFFITT
LPTRCRLEIIEDYYDAIAKNISEEYISYGGEKKILAIQGALEKALRWASGESFIELSNHKFIRMKRKLMYVSADATKWSPGDNSAKFRRFTSMLHNGLPNNKLKNCVIDALKQVY
KTDFFMSRKLRNYIDSMESLDPHIKQFLDFFPDGHHGEVKGNWLQGNLNKCSSLFGVAMSLLFKQVWTNLFPELDCFFEFAHHSDDALFIYGYLEPVDDGTDWFLFVSQQIQAGH
LHWFSVNTEMWKSMFNLHEHILLLGSIKISPKKTTVSPTNAEFLSTFFEGCAVSIPFVKILLGSLSDLPGLGYFDDLAAAQSRCVKALDLGASPQVAQLAVALCTSKVERLYGTA
PGMVNHPAAYLQVKHTDTPIPLGGNGAMSIMELATAGIGMSDKNLLKRALLGYSHKRQKSMLYILGLFKFLMKLSDETFQHERLGQFSFIGKVQWKIFTPKSEFEFADMYTSKFL
ELWSSQHVTYDYIIPKGRDNLLIYLVRKLNDPSIVTAMTMQSPLQLRFRMQAKQHMKVCRLDGEWVTFREVLAAANSFAENYSATSQDMDLFQTLTSCTFSKEYAWKDFLNGIHC
DVIPTKQVQRAKVARTFTVREKDQIIQNSIPAVIGYKFAVTVEEMSDVLDTAKFPDSLSVDLKTMKDGVYRELGLDISLPDVMKRIAPMLYKSSKSRVVIVQGNVEGTAEAICRY
WLKSMSLVKTIRVKPHKEVLQAVSIFNRKEDIGQQKDLAALKLCIEVWRWCKANSAPYRDWFQALWFEDKTFSEWLDRFCRVGVPPIDPEIQCAALMIADIKGDYSVLQLQANRR
AYSGKQYDAYCVQTYNEVTKLYEGDLRVTFNFGLDCARLEIFWDKKAYILETSITQKHVLKIMMDEVSKELIKCGMRFNTEQVQGVRHMVLFKTESGFEWGKPNIPCIVYKNCVL
RTSLRTTQAINHKFMITIKDDGLRAIAQHDEDSPRFLLAHAFHTIRDIRYQAVDAVSNVWFIHKGVKLYLNPIISSGLLENFMKNLPAAIPPAAYSLIMNRAKISVDLFMFNDLL
KLINPRNTLDLSGLETTGDEFSTVSSMSSRLWSEEMSLVDDDEELDDEFTIDLQDVDFENIDIEADIEHFLQDESSYTGDLLISTEETESKKMRGIVKILEPVRLIKSWVSRGLS
IEKVYSPVNIILMSRYISKTFNLSTKQVSLLDPYDLTELESIVRGWGECVIDQFESLDREAQNMVVNKGICPEDVIPDSLFSFRHTMVLLRRLFPQDSISSFY

Coverage: 68.02%

Supplementary Figure 2 Nanoscale liquid chromatography coupled to tandem mass spectrometry analysis (LC-MS/MS) of trypisinized bands extracted from an SDS-PAGE gel of HTNV-L purified sample

a List of the five most abundant proteins quantified by nano LC-MS/MS in the 250kDa and the 150kDa bands. The number of identified peptides (pep), the spectral counts (SC), the intensity-based absolute quantification (iBAQ) and the rank are indicated. Except from HTNV-L, all the proteins listed originate from the expression in *Trichoplusia ni*.

b HTNV-L sequence with the identified peptides highlighted in grey in the 150kDa (top) and the 250kDa (bottom) SDS-PAGE bands.

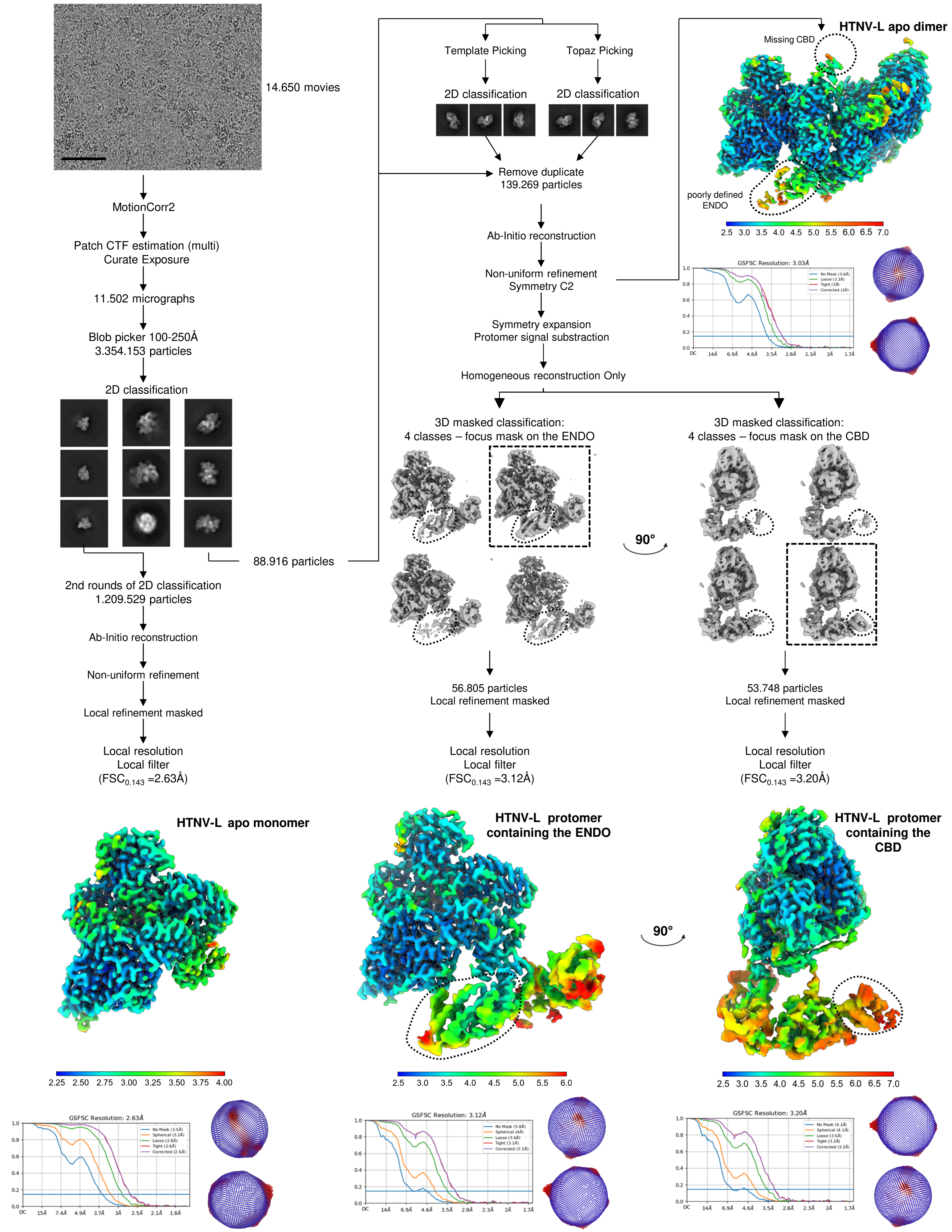

Supplementary Figure 3 Image processing strategy to obtain apo monomeric and dimeric HTNV-L structures

A representative image of HTNV-L apo is displayed. The scale bar corresponds to 50 nm. The experiment was repeated two times with similar results. The image processing workflow including 2D class averages, 3D class averages and the final reconstructions are displayed. Regions used for masking are indicated with a dotted line. 3D class averages chosen for further processing are surrounded by a rectangle dotted line. Electron density maps are colored according to their local resolution. Gold-standard Fourier Shell Correlation curves (FSC) and angular distributions of particles used in the final reconstructions are displayed.

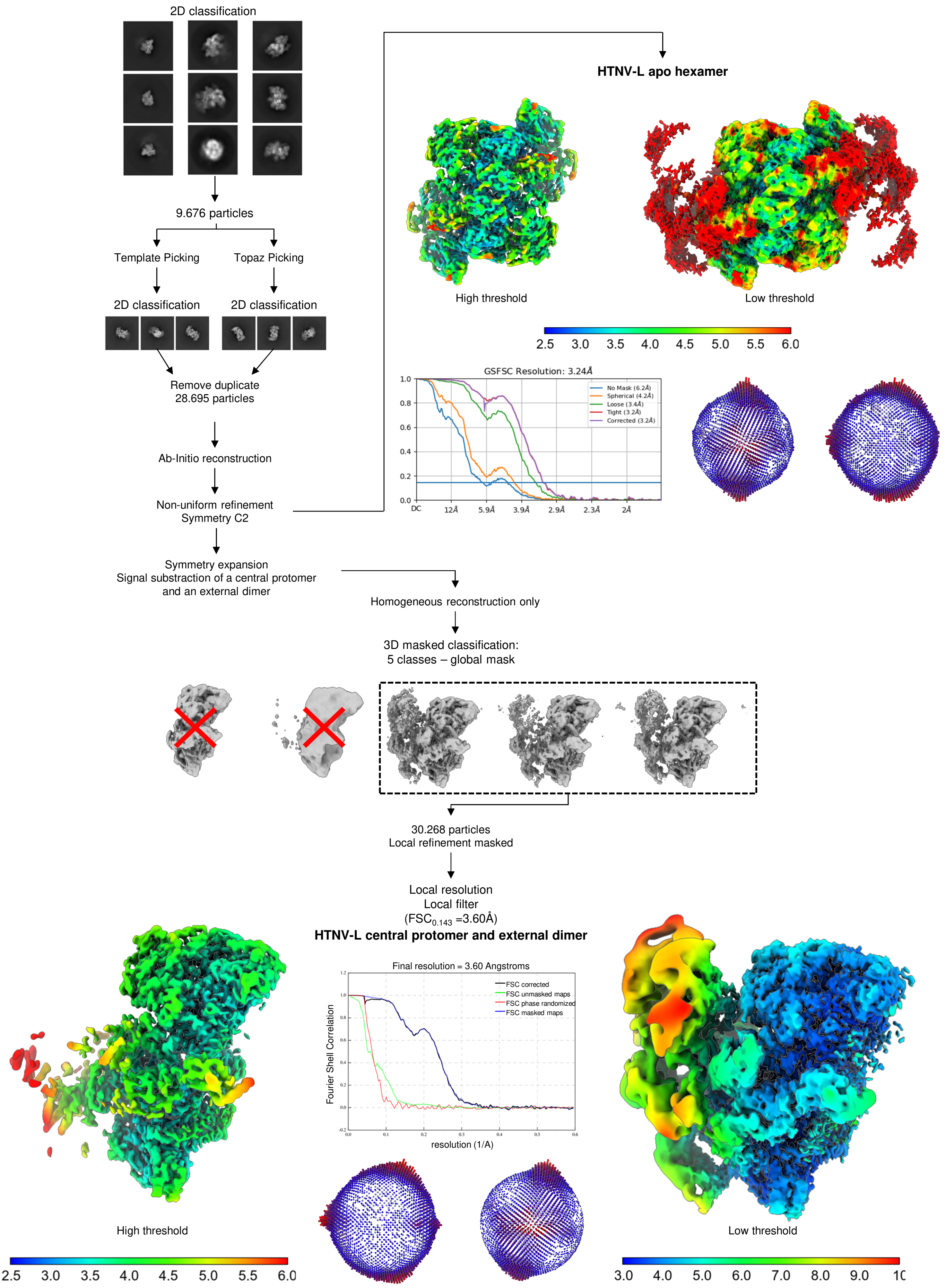

Supplementary Figure 4 Image processing strategy to obtain apo hexameric HTNV-L structure

The strategy is displayed from the 1st round of 2D class averages also shown in **Supplementary Figure 3.** 2D class averages, 3D class averages and the final reconstructions are displayed. 3D class averages chosen for further processing are surrounded by a rectangle dotted line. Electron density maps are colored according to their local resolution. Fourier Shell Correlation curves (FSC) and angular distributions of particles used in the final reconstructions are displayed.

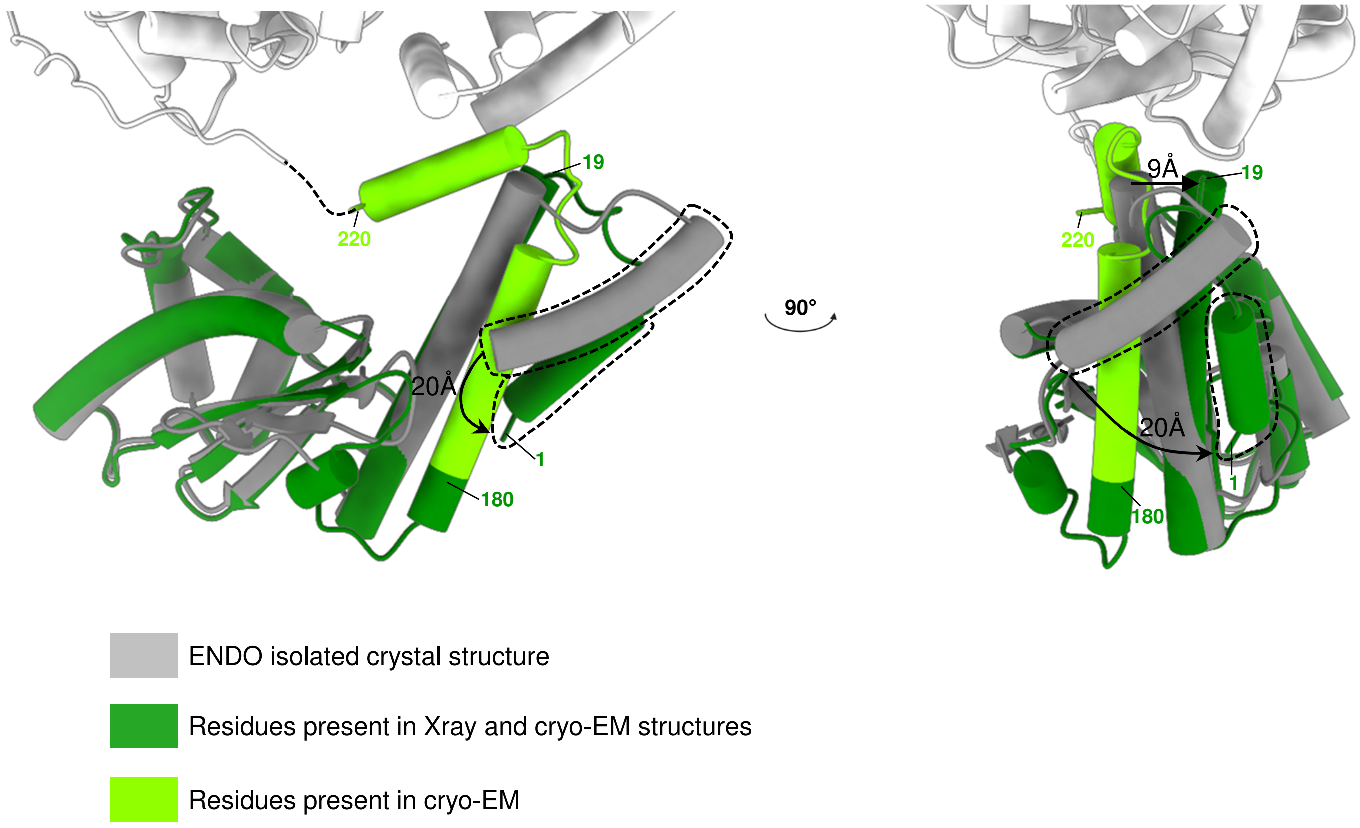

Supplementary Figure 5 Comparison of HTNV ENDO structures in the isolated domain context versus the full-length HTNV-L context

Superposition of HTNV ENDO structures: (i) in the isolated domain context from X-ray crystallography colored in gray, (ii) in the full-length HTNV-L context from cryo-EM colored in green. In the cartoon representation, residues in dark green are the ones present in both structures, and residues in light green are the ones present only in the cryo-EM HTNV-L full-length structure. The rotation amplitude is indicated. The α-helix comprising residues 1 to 18 that moves significantly is surrounded by a dotted line.

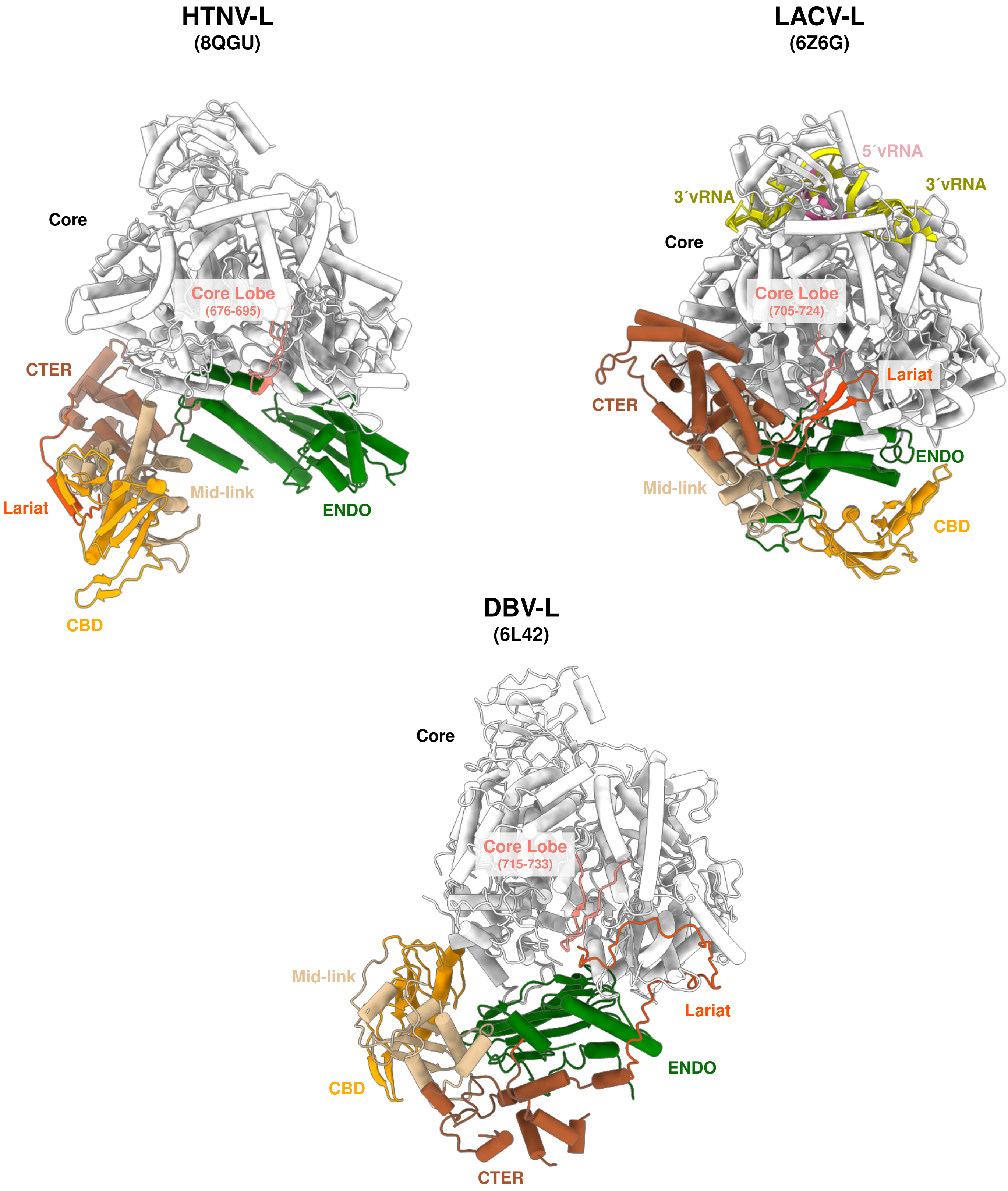

Supplementary Figure 6 Comparison of lariat/β-hairpin strut protrusions in Bunyaviruses

HTNV-L, LACV-L and DBV-L are shown as cartoon and colored as in **Fig.2**. The β-hairpin strut of LACV-L and the lariat of HTNV-L and DBV-L are displayed in red. The β-hairpin present on the core-lobe of the three polymerases is colored in salmon.

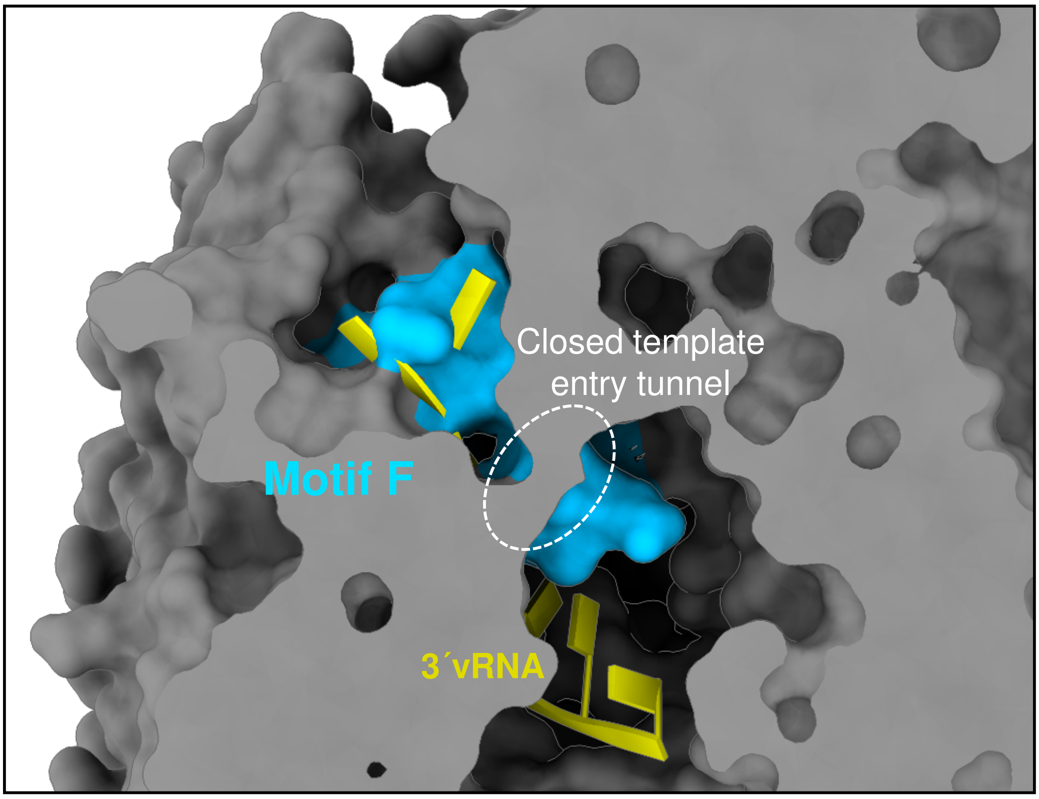

Supplementary Figure 7 Closure of the template entry tunnel by the motif F in HTNV-L apo

HTNV-L apo core is colored as a white surface and is clipped to visualize the 3´vRNA template entry tunnel. The location of the motif F is shown as a blue surface. The 3′vRNA end that originates from HTNV-L in pre-initiation (PDB 8C4U) is displayed as a yellow cartoon.

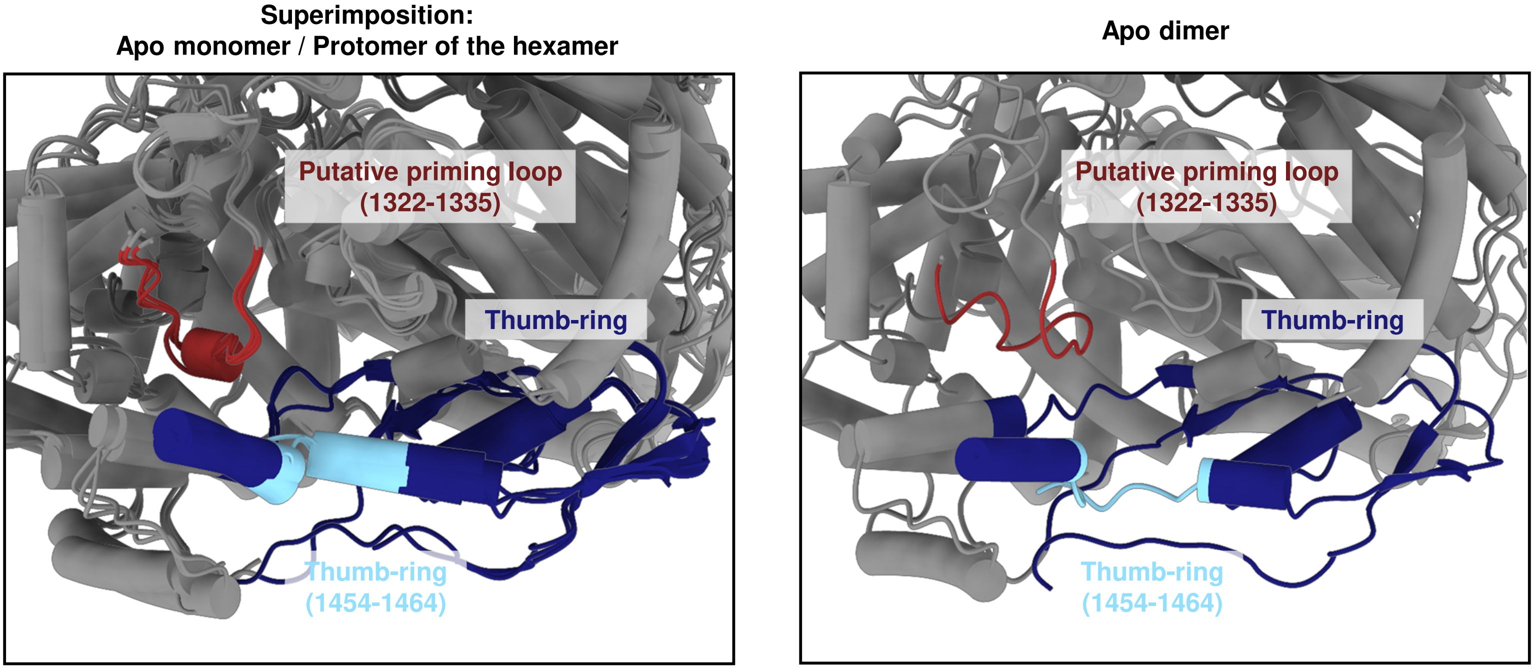

Supplementary Figure 8 Comparison of the putative priming loop positions in the different HTNV-L apo oligomers

Zoom on the putative priming loop (or template exit plug) colored in dark red and thumb-ring colored in dark blue. The putative priming loop and thumb-ring region 1454-1464, that is colored in light blue, change their organization in the isolated apo HTNV-L dimer (right panel) compared to apo HTNV-L monomer and hexamer (left panel).

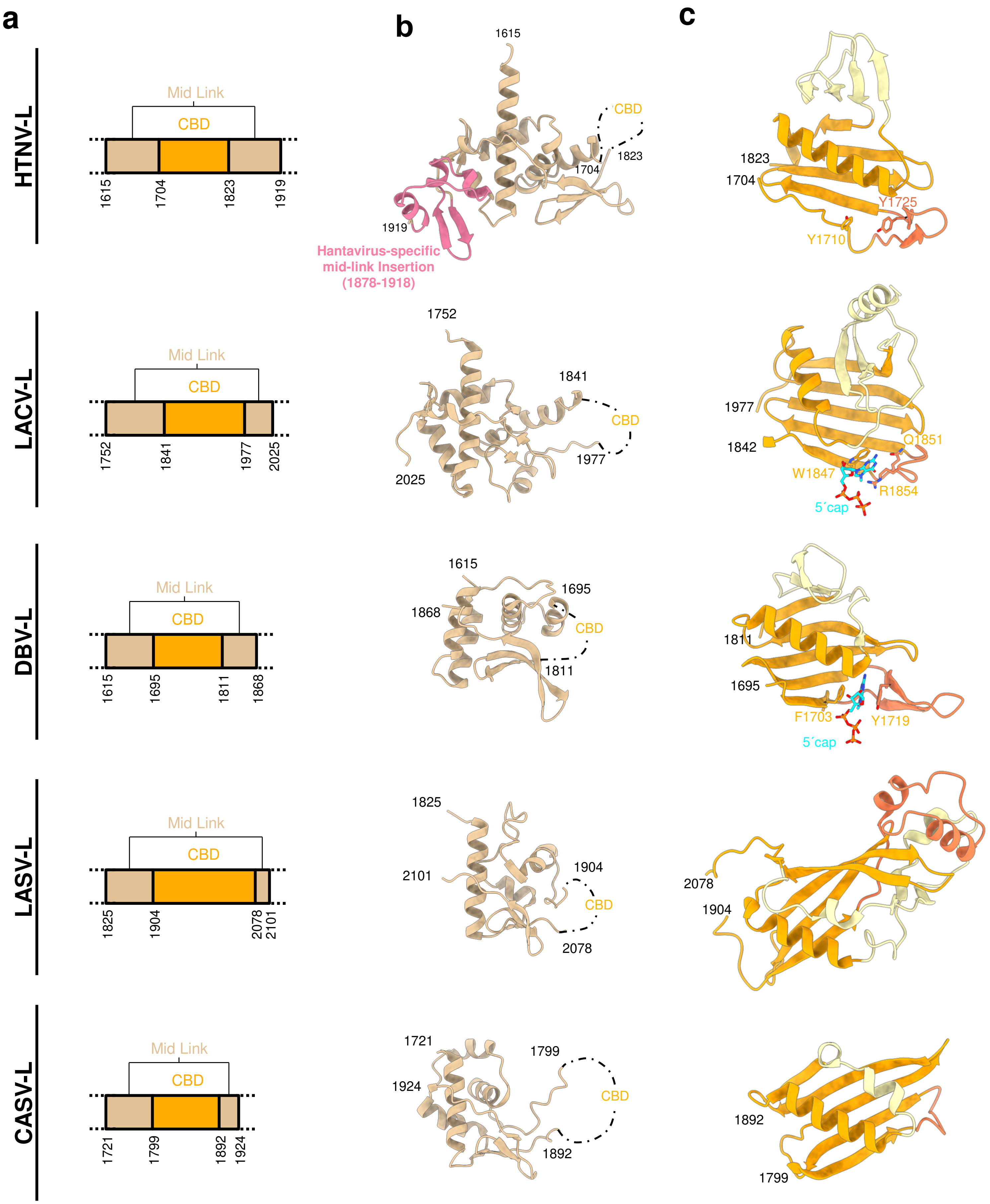

Supplementary Figure 9 Mid-link and CBD organization in Bunyaviruses

a Schematic representation of HTNV-L domain structure zoomed on the mid-link and the CBD.

b Mid-link domains from different Bunyaviruses colored in beige. The hantavirus-specific insertion is colored in light pink.

c CBD domains from different sNSV. The central β-sheet and α-helix that are common to all Bunyavirus CBD are shown in orange cartoon. The β-hairpin insertion that is likely to be essential for cap binding is shown in dark orange. The region specific to each bunyavirus family is shown in light yellow. For LACV-L and DBV-L, the cap is shown as blue stick and residues that stack the cap are displayed as orange sticks. For HTNV-L and LASV-L, the residues that are likely to stack the cap due to their positioning are shown as sticks and are labeled. CASV-L is missing the β-hairpin insertion and cannot stack the cap.

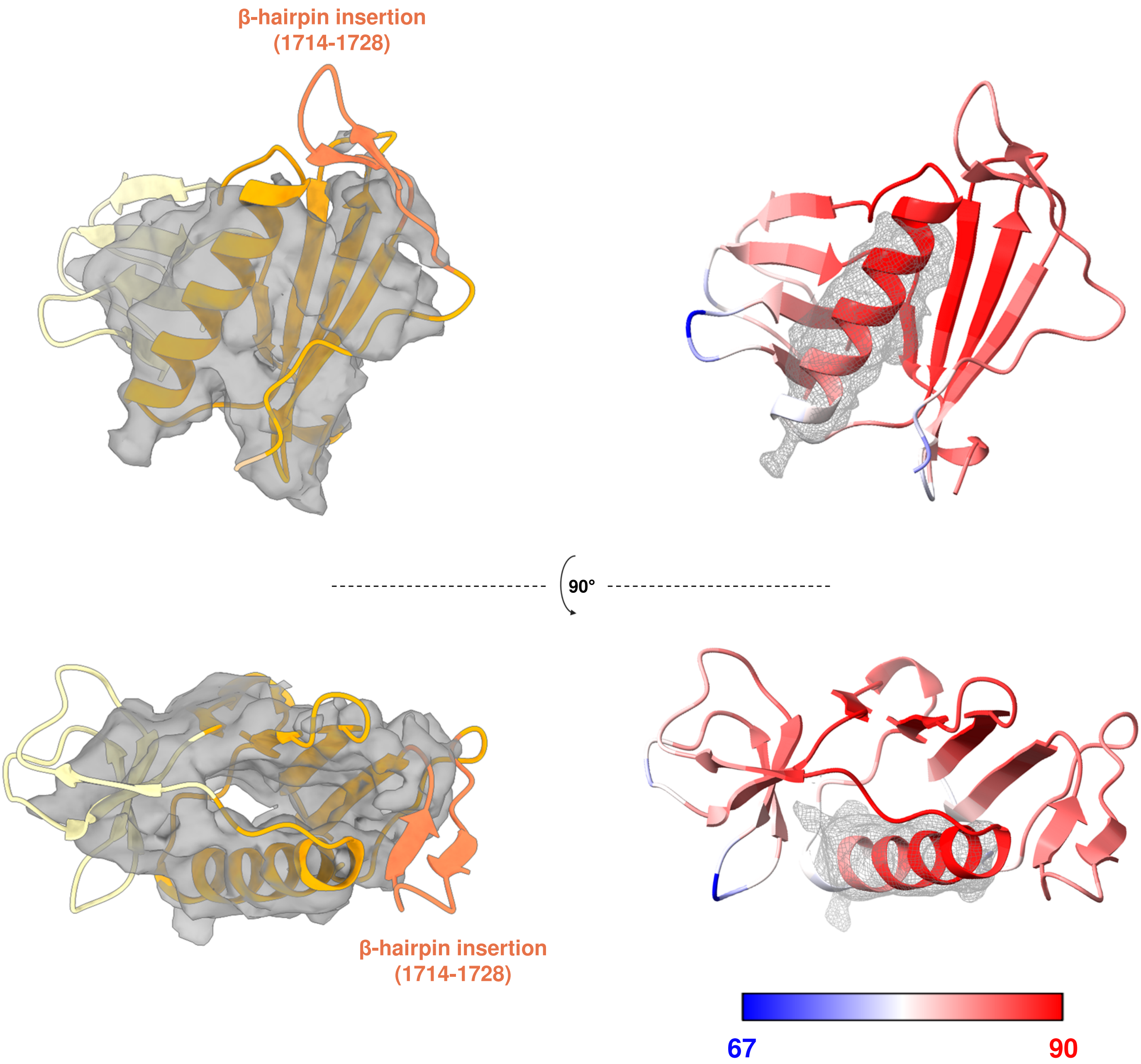

Supplementary Figure 10 CBD envelope in HTNV-L apo dimer cryo-EM map

On the left, the HTNV-L CBD model is shown as cartoon and colored as in **Fig. 2.** The corresponding cryo-EM density is shown as transparent gray surface. Although the resolution of this part remains low, the global fit is unambiguous as the global shape corresponds, with the central β-sheet and α-helix and the hantavirus-specific insertion that can be positioned in density. The β-hairpin insertion is not visible due to a too large flexibility.

On the right, the model is colored according to Alphafold pLDDT criteria. The electron density that corresponds to the α-helix is shown at a different thresholds to show that it enables an unambiguous overall positioning of the CBD.

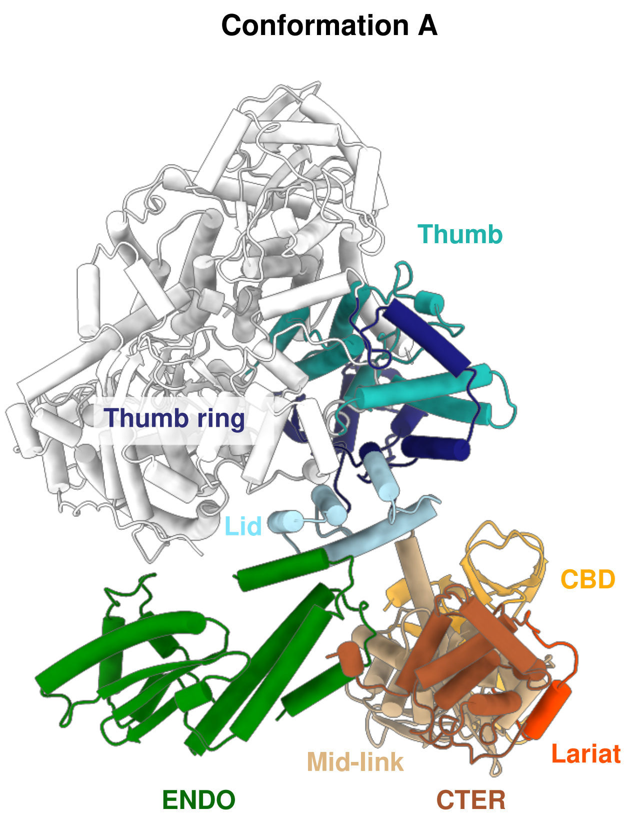

Supplementary Figure 11 Interaction of the ENDO and the C-terminal domains with the polymerase cores in HTNV-L conformers A and B
Polymerase cores of HTNV-L conformers A and B are displayed as white cartoon. The domains that interact with either the ENDO or the C-terminal domains are colored: the lid in light blue, the thumb in light sea green and the thumb-ring in midnight blue.

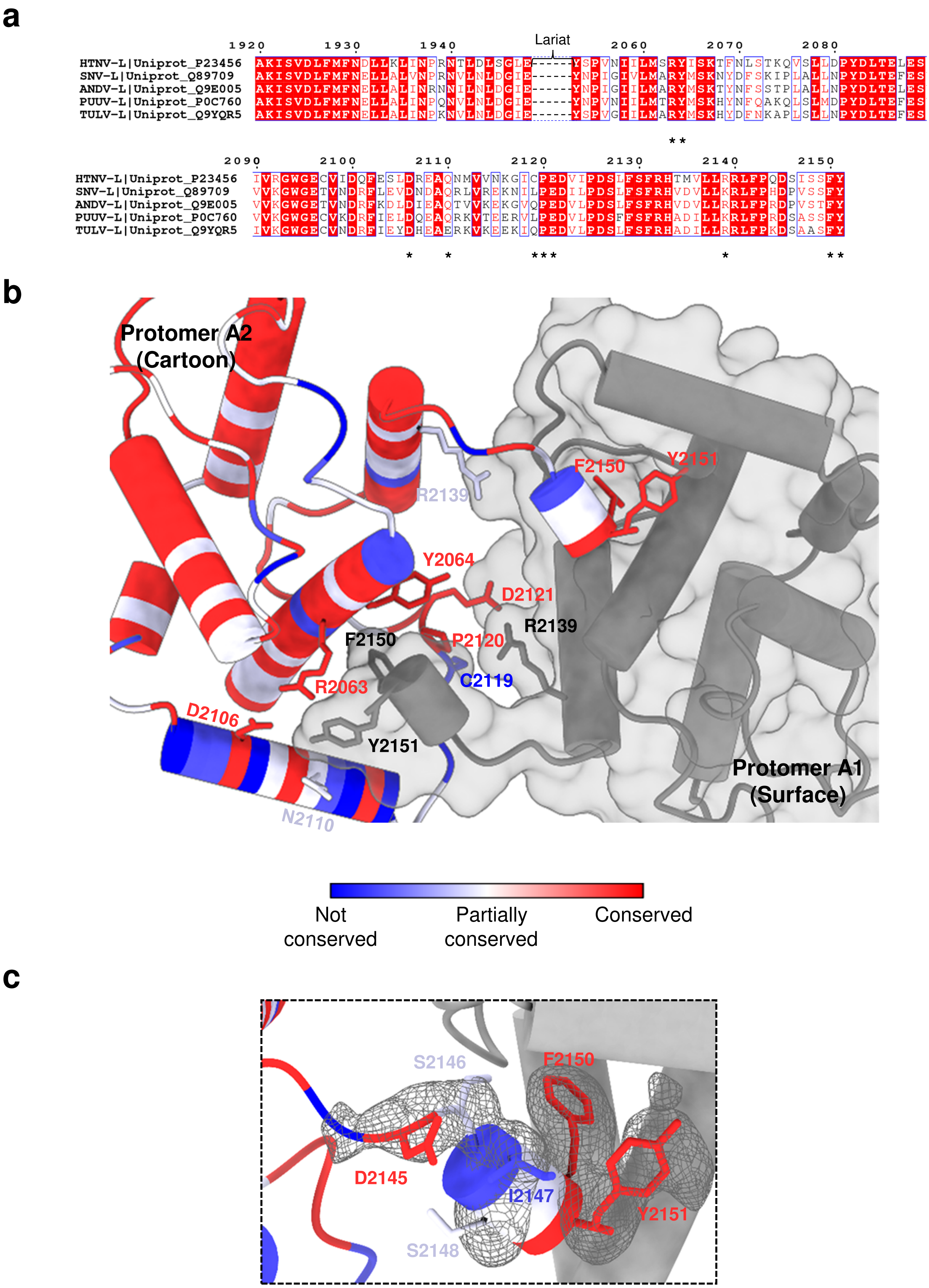

Supplementary Figure 12 Conservation of the residues involved in the interactions between HTNV-L CTER domains

a Multiple alignment displaying the CTER domain. Residues involved in CTER-CTER interaction are indicated with stars.

b Zoom on the CTER domains of HTNV-L apo symmetric dimer. For clarity, protomer A1 is shown as a grey cartoon with transparent surface and protomer A2 is shown as cartoon with the amino acid involved in interactions colored according to the sequence conservation, from red if conserved to dark blue if not conserved. Residues involved in CTER-CTER interactions are shown as sticks and are labeled.

c Zoom on the model of the extreme HTNV-L C-terminal loop with the electron density displayed as mesh. Protomer and amino acid are colored as in **b**.

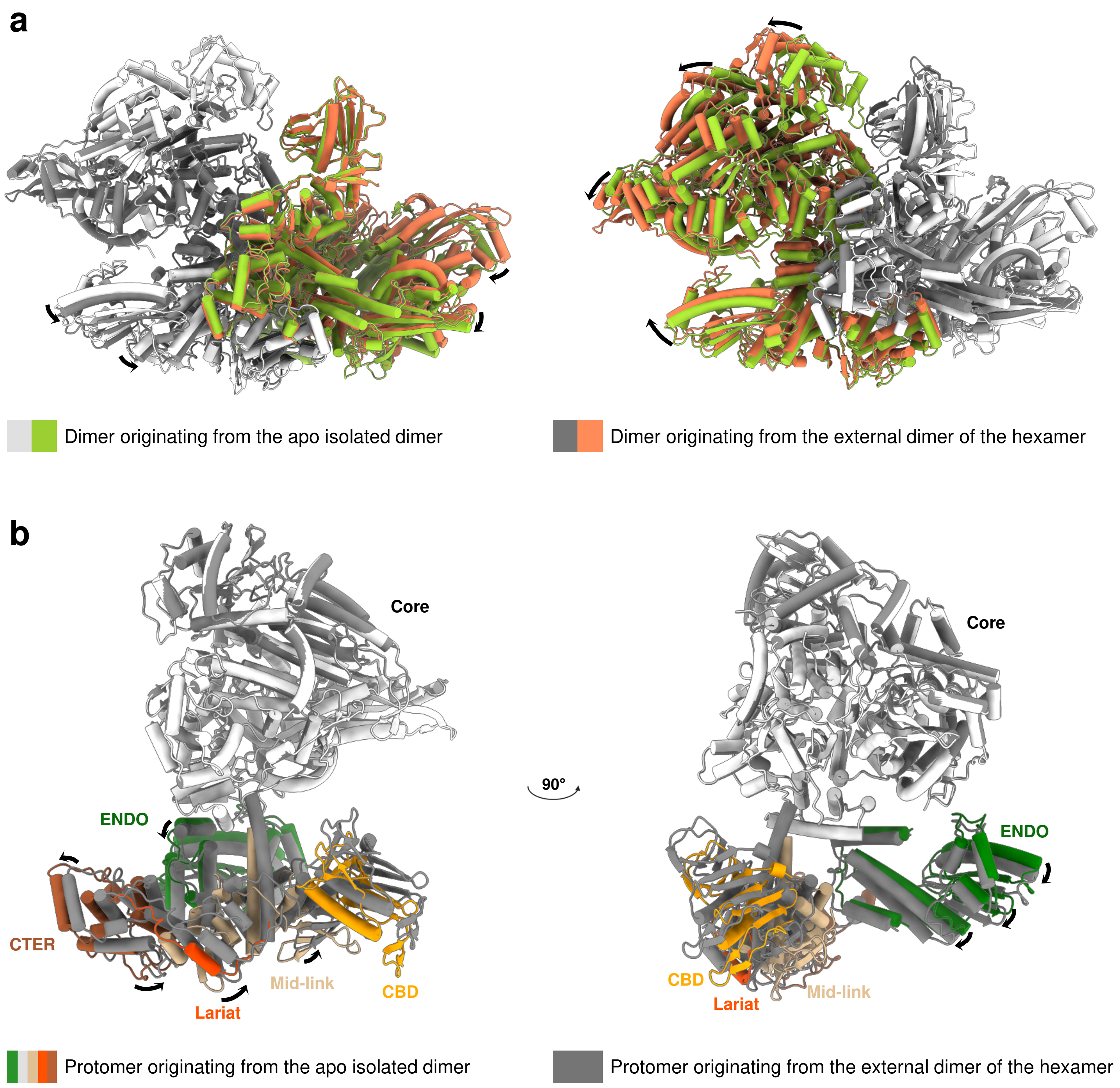

Supplementary Figure 13 Comparison of dimers A in HTNV-L apo isolated dimer and HTNV-L external dimer of the hexamer

a superimposition of HTNV-L dimers A originating from the apo isolated dimer (colored in white and green) and from the external dimer of the hexamer (colored in grey and coral). A protomer originating from each dimer is superimposed and the relative rotation of the second protomer is indicated.

b superimposition of HTNV-L protomer originating from the apo isolated dimer and from the external dimer of the hexamer, with their core respectively colored in white and grey. The small rotations of the ENDO and the domains of the C-terminal region, that are rigidly fitted in the density, are indicated.

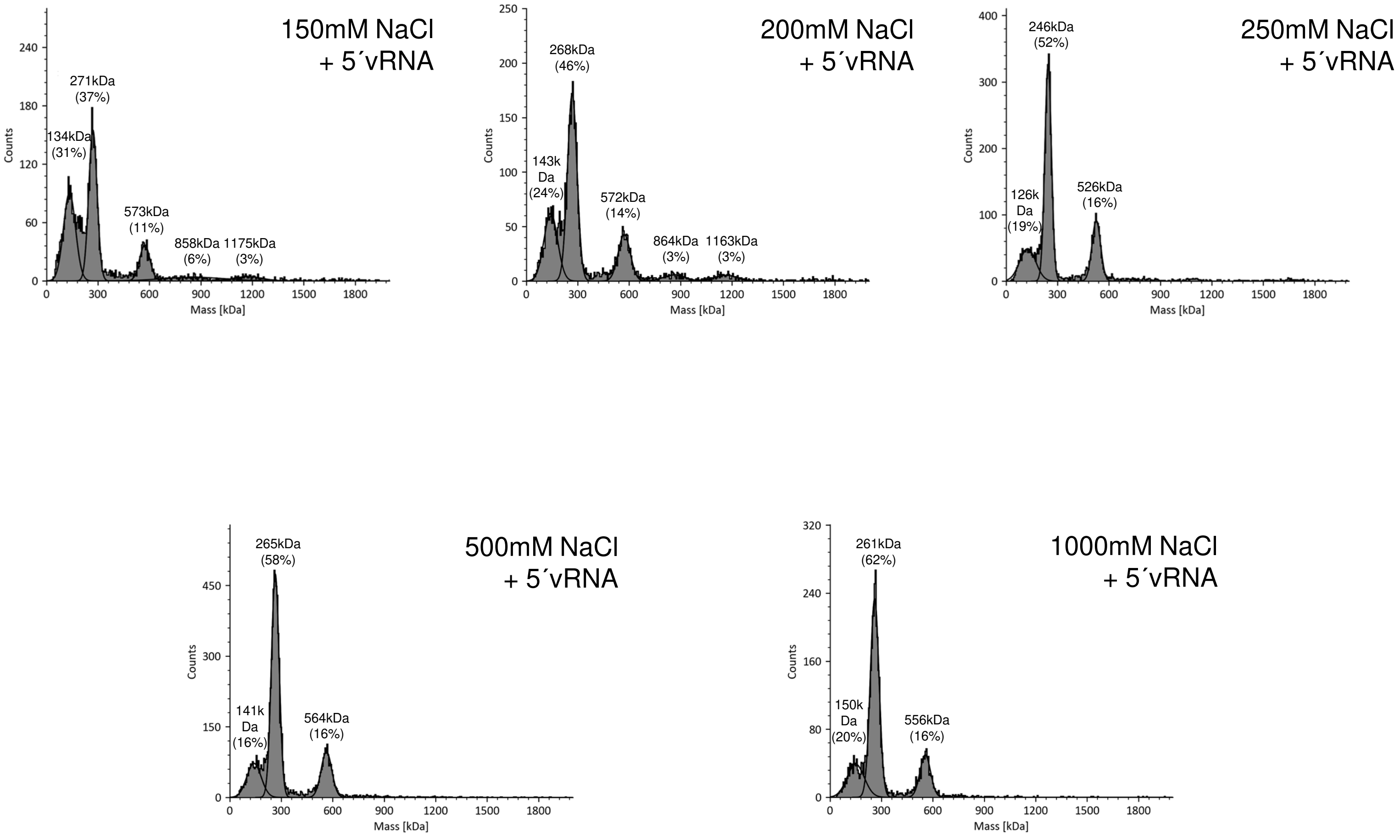

Supplementary Figure 14 Mass photometry of HTNV-L in presence of 5′vRNA at different ionic strength concentrations

The HTNV-L:5′vRNA ratio is 1:10. The buffers used for the measurements contain 30 mM HEPES, 5 mM TCEP and a concentration of NaCl that varies between 150 mM and 1M as indicated. The molecular mass in kDa and the percentage of each species are indicated.

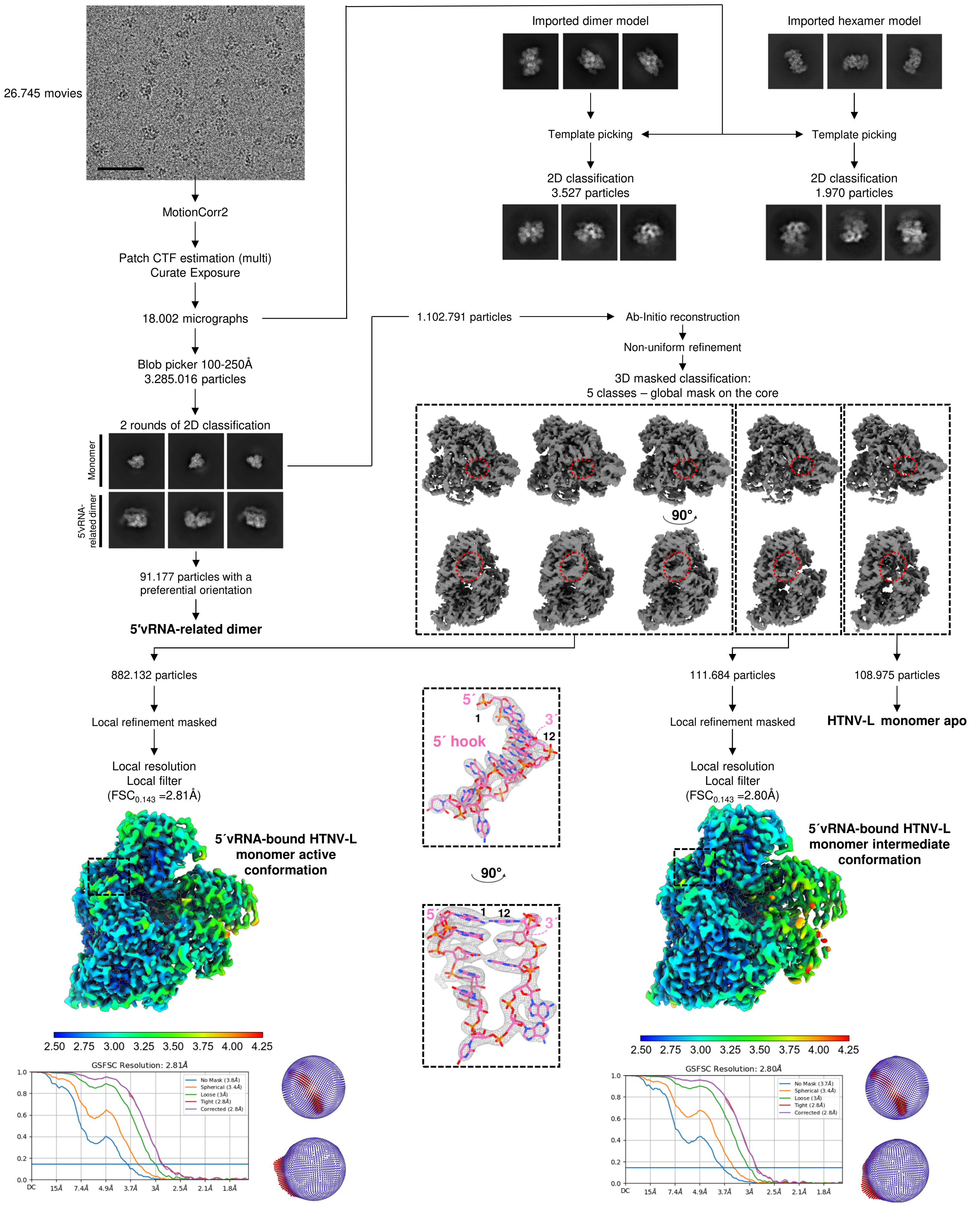

Supplementary Figure 15 Image processing strategy of the HTNV-L dataset collected in presence of 5′vRNA

A representative micrograph of HTNV-L collected in presence of 5′vRNA is displayed. The experiment was repeated two times with seminal results. The scale bar corresponds to 50 nm. Picking on the dataset did not reveal any symmetric dimers or hexamers but revealed the presence of monomers and another type of 5′vRNA-related dimers. Import of symmetric dimers and hexamers from the apo HTNV-L dataset followed by template picking selected only a very low number of symmetric dimers and hexamers, preventing further processing. The image processing workflow including 2D class averages, 3D class averages and the final reconstructions of monomeric 5′vRNA-bound HTNV-L are displayed. Electron density maps are colored according to their local resolution. Fourier Shell Correlation curves (FSC) and angular distributions of particles used in the final reconstructions are displayed. A zoom on the 5′vRNA is shown with the model displayed as stick and the electron density displayed as mesh.

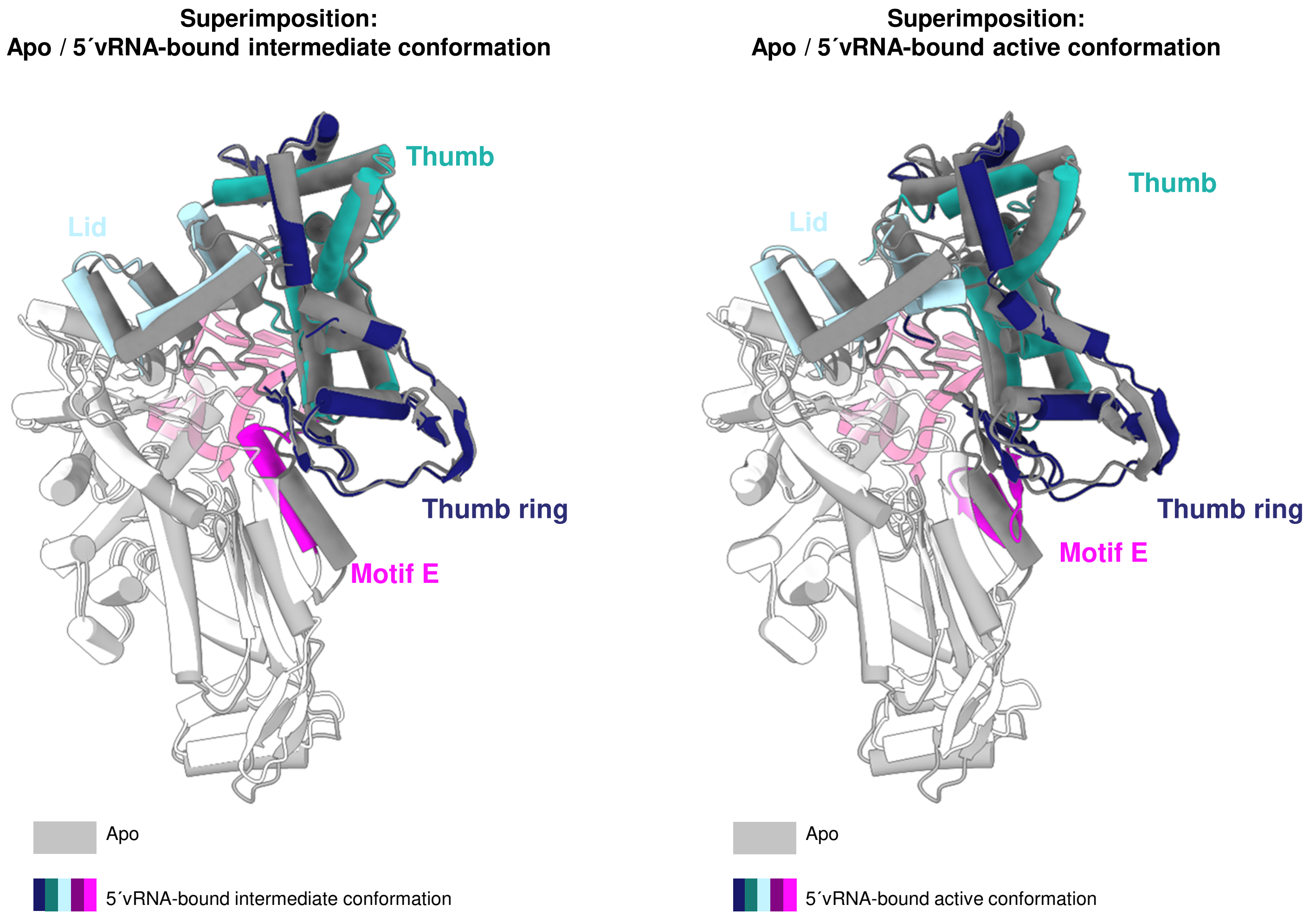

Supplementary Figure 16 Comparison of HTNV-L polymerase cores

HTNV-L apo monomeric structure is used as reference for superimposition and is displayed in white. It is superimposed with 5′vRNA-bound HTNV-L intermediate conformation (left) and 5′vRNA-bound active conformation (right). They are displayed as cartoon and colored in grey excepting their 5′vRNA, thumb, thumb-ring, lid and motif E that are respectively colored in light pink, light sea green, midnight blue, light blue and magenta.

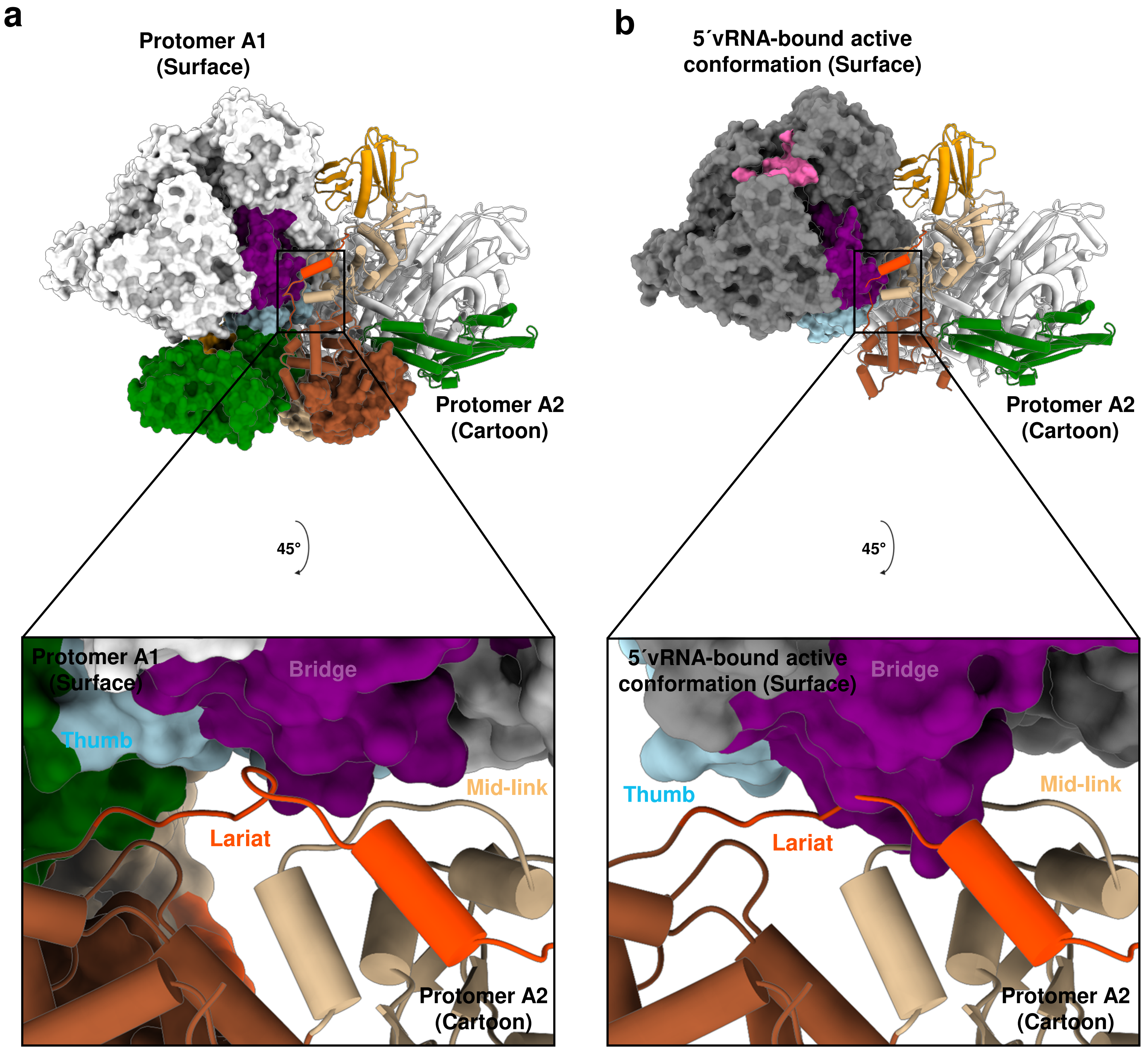

Supplementary Figure 17 Superimposition the 5′vRNA-bound monomeric HTNV-L structure onto the apo HTNV-L symmetric dimer structure reveals clashes

a Structure of apo HTNV-L symmetric dimer with protomer 1 shown as surface and protomer 2 shown as cartoon. The domains are colored as in **Fig. 2** except the bridge and the thumb of protomer 1 that are respectively displayed in purple and in light blue. The interaction between the lariat and the bridge is visible on the zoom displayed at the bottom.

b When superimposing the core of 5′vRNA-bound HTNV-L monomer on HTNV-L apo dimer protomer 1, clashes appear with HTNV-L apo dimer protomer 2. HTNV-L apo dimer protomer 1 is not shown, HTNV-L apo dimer protomer 2 is shown as in **a**, 5′vRNA-bound HTNV-L core is shown as a grey surface with the 5′vRNA, the bridge domain and the thumb domain respectively colored in light pink, purple and in light blue. The zoom at the bottom identifies clashes between the lariat of HTNV-L apo dimer protomer 2 and the bridge of 5′vRNA-bound HTNV-L core.

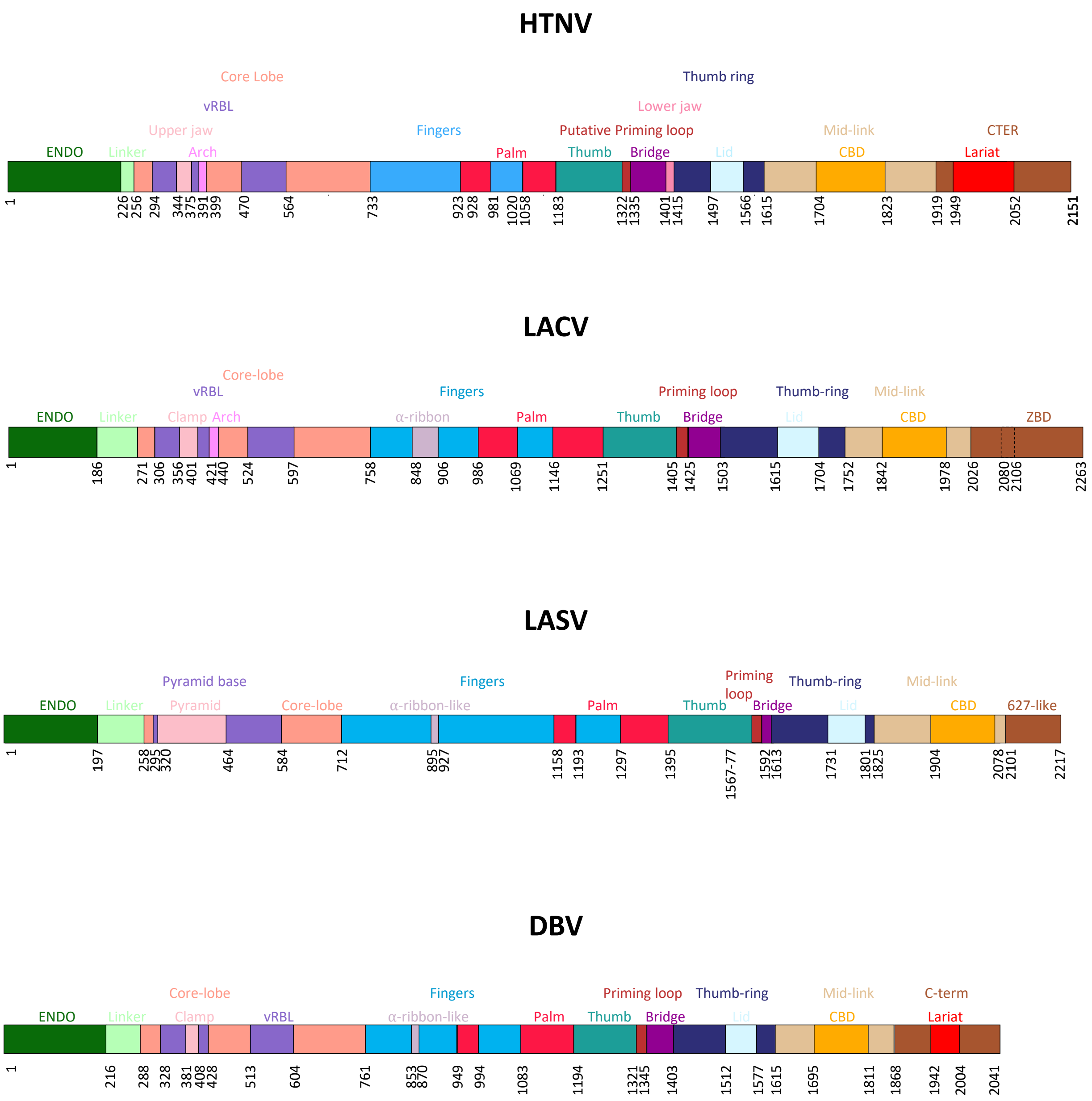

Supplementary Figure 18 Schematic representation of Bunyaviruses domain structure

Each domain is colored and its extreme residues are numbered.

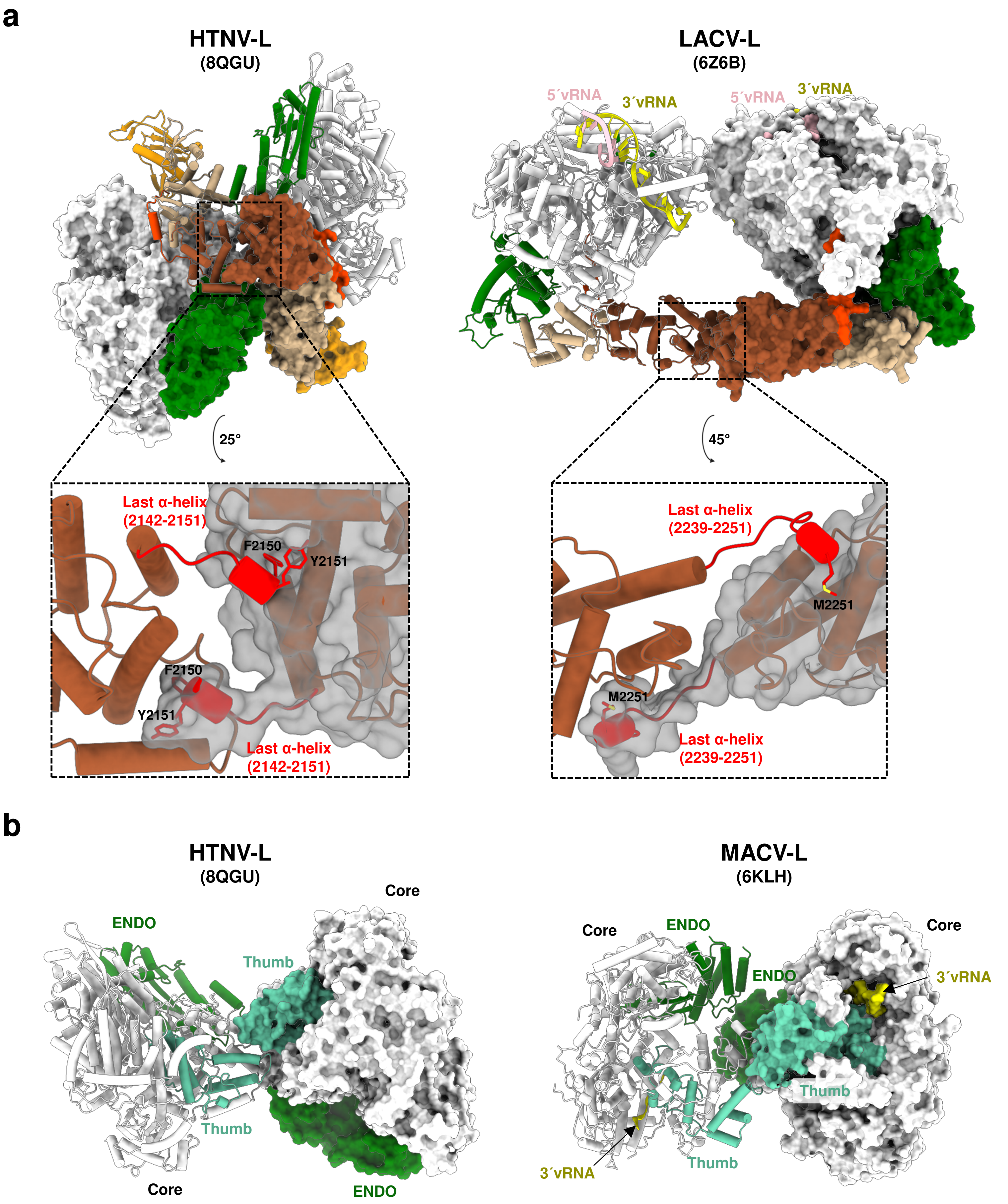

Supplementary Figure 19 Comparison of apo HTNV-L symmetric dimer with other sNSV polymerase symmetric dimers

a HTNV-L and LACV-L symmetric dimers colored as in **Fig. 4**. One protomer is shown as surface and the other as cartoon. LACV-L is bound to 5′ and 3′vRNA ends that are respectively shown as light pink and yellow cartoons. At the bottom, zoom on the C-terminal ends, with the surface of one protomer shown. The C-terminal extremities that swap in the other protomer are shown in red, with the conserved hydrophobic C-terminal residues shown as sticks.

b HTNV-L and MACV-L symmetric dimers with one protomer shown as cartoon and the second displayed as a surface. The cores are shown in white with the exception of the thumbs that are colored in light sea green. The ENDO are colored in forest green. The 3′vRNA that is bound to MACV-L dimer is shown in yellow.

Supplementary Table 1. Cryo-EM data collection, refinement and validation statistics of apo HTNV-L 3D structures

	HTNV-L apo monomer	HTNV-L apo symmetric dimer			HTNV-L apo symmetric hexamer	
	PDB 8QE5<br>EMD-18343	PDB 8QGU<br>EMD-18391 (composite map)			PDB 8QHD<br>EMD-18408<br>(composite map)	
		EMD-18392 (overall dimer)	EMD-18393 (refine ENDO)	EMD-18394 (refine CBD)	EMD-18406 (overall hexamer)	EMD-18405 (one central and two external protomers)
**Data collection and processing**						
Microscope	Thermo Fisher Scientific Krios					
Camera	Gatan K3					
Magnification	105 000					
Voltage (kV)	300					
Electron exposure (e-/A2)	40					
Defocus range (µm)	-0.8 to -2.0					
Pixel size (Å)	0.839					
Initial/Final micrographs (no.)	14650/11502					
Symmetry imposed	C1	C2	C1	C1	C2	C1
Final particles (no.)	142.391	139.269	56.805	53.788		
Map resolution (Å)	2.6	3.0	3.1 (around 4 Å for the ENDO	3.2 (around 8 Å for the CBD)	3.2	3.6
FSC threshold	0.143	0.143			0.143	
Map resolution range (Å)	2.25-4.0	2.5-7.0	2.5-6.0	2.5-7.0	2.5-10.0	2.5-10.0
**Refinement**						
Model resolution (Å) 0.5 FSC threshold	2.6	3.2				3.3
Map sharpening B factor (Å^2)	-80	-80			-80	-80
Model composition					Two internal protomers	One internal protomer and one external protomer
Protein residues	1340	4064			3746	3872
B-factor (Å^2, min-max (mean))	0.00-61.72 (19.56)	23.64-257.21-(76.92)			56.56-486.5-(157.97)	7.76-246.25 (94.94)
R.m.s deviations						
Bond lengths (Å)	0.003	0.002			0.002	0.004
Bond angles (°)	0.513	0.503			0.517	0.6
**Validation**						

MolProbity score	1.95	1.63	1.81	1.89
Clashscore	7.16	7.82	9.99	10.79
Poor rotamers (%)	3.02	0.00	0.42	0.41
**Ramachandran plot**				
Favored (%)	96.83	96.73	95.79	95.17
Allowed (%)	3.17	3.27	4.05	4.80
Disallowed (%)	0	0	0.16	0.03

Supplementary Table 2. Cryo-EM data collection, refinement and validation statistics of 5'-bound HTNV-L 3D structures

	**HTNV-L 5'-bound "intermediate" monomer**	**HTNV-L 5'-bound "activated" monomer**
	PDB 8QGT<br>EMD-18390	PDB 8QH3<br>EMD-18397
**Data collection and processing**		
Microscope	Thermo Fisher Scientific Krios	
Camera	Gatan K3	
Magnification	105 000	
Voltage (kV)	300	
Electron exposure (e-/A2)	40	
Defocus range (μm)	-0.8 to -2.0	
Pixel size (Å)	0.839	
Initial/Final micrographs (no.)	26745/18002	
Symmetry imposed	C1	
Final particles (no.)	882.132	111.684
Map resolution (Å)	2.8	2.8
FSC threshold	0.143	0.143
Map resolution range (Å)	2.50-4.25	2.5-4.25
**Refinement**		
Model resolution (Å) 0.5 FSC threshold	2.8	2.8
Map sharpening B factor (Å^2)	-80	-80
Model composition		
Protein residues	1289	1309
Nucleotide residues	12	11
Ligands	0	1
Water	0	0
B-factor (Å^2, min-max (mean))		
Protein	0/64.18 (18.12)	0/37.47 (8.45)
Nucleotides	0/60.81 (12.44)	0/37.83 (10.13)
Ligands	0	10.34/10.34 (10.34)
R.m.s deviations		
Bond lengths (Å)	0.003	0.003
Bond angles (°)	0.502	0.506
**Validation**		
MolProbity score	1.78	1.73
Clashscore	7.14	7.49
Poor rotamers (%)	2.11	1.55
Ramachandran plot		
Favored (%)	97.24	96.99
Allowed (%)	2.76	2.93
Disallowed (%)	0	0
