# Supplementary material for: Structural characterization of the oligomerization of full-length Hantaan virus polymerase into symmetric dimers and hexamers
Source: Nat Commun. 2024 Mar 13;15:2256. doi: 10.1038/s41467-024-46601-4 (PMC10937945; doi:10.1038/s41467-024-46601-4)

Supplementary Data (1/3)

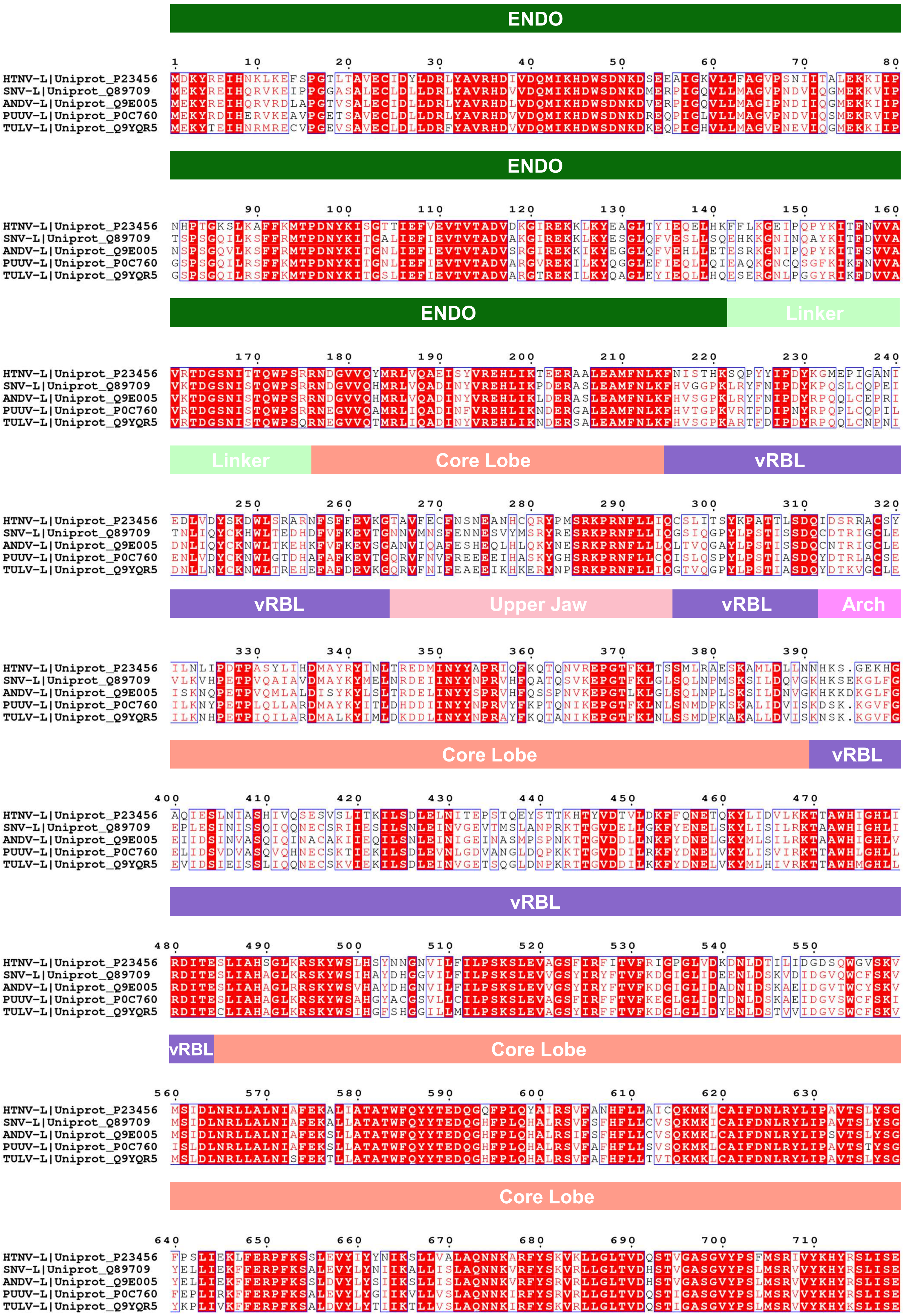

Supplementary Data (2/3)

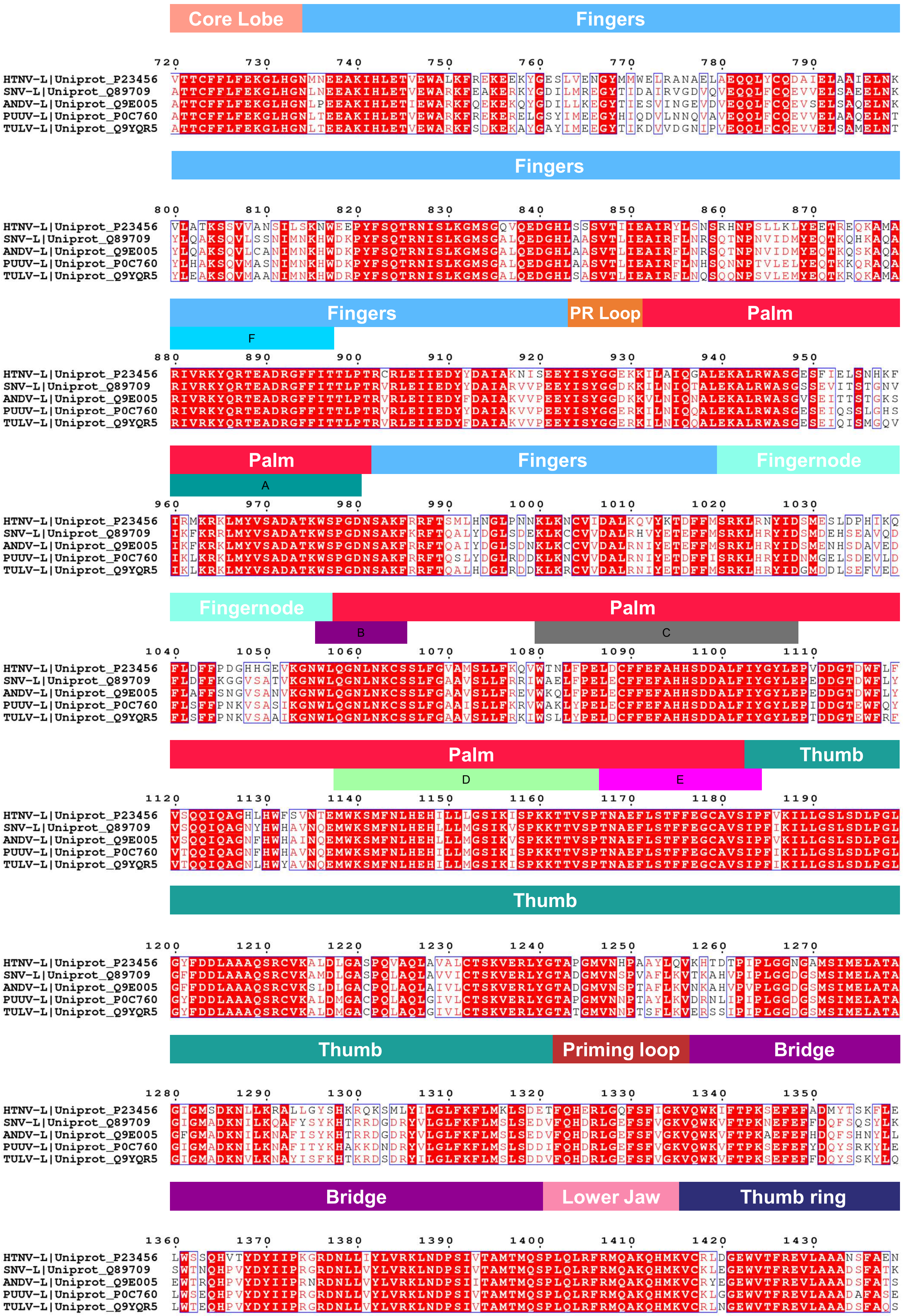

Supplementary Data (3/3)

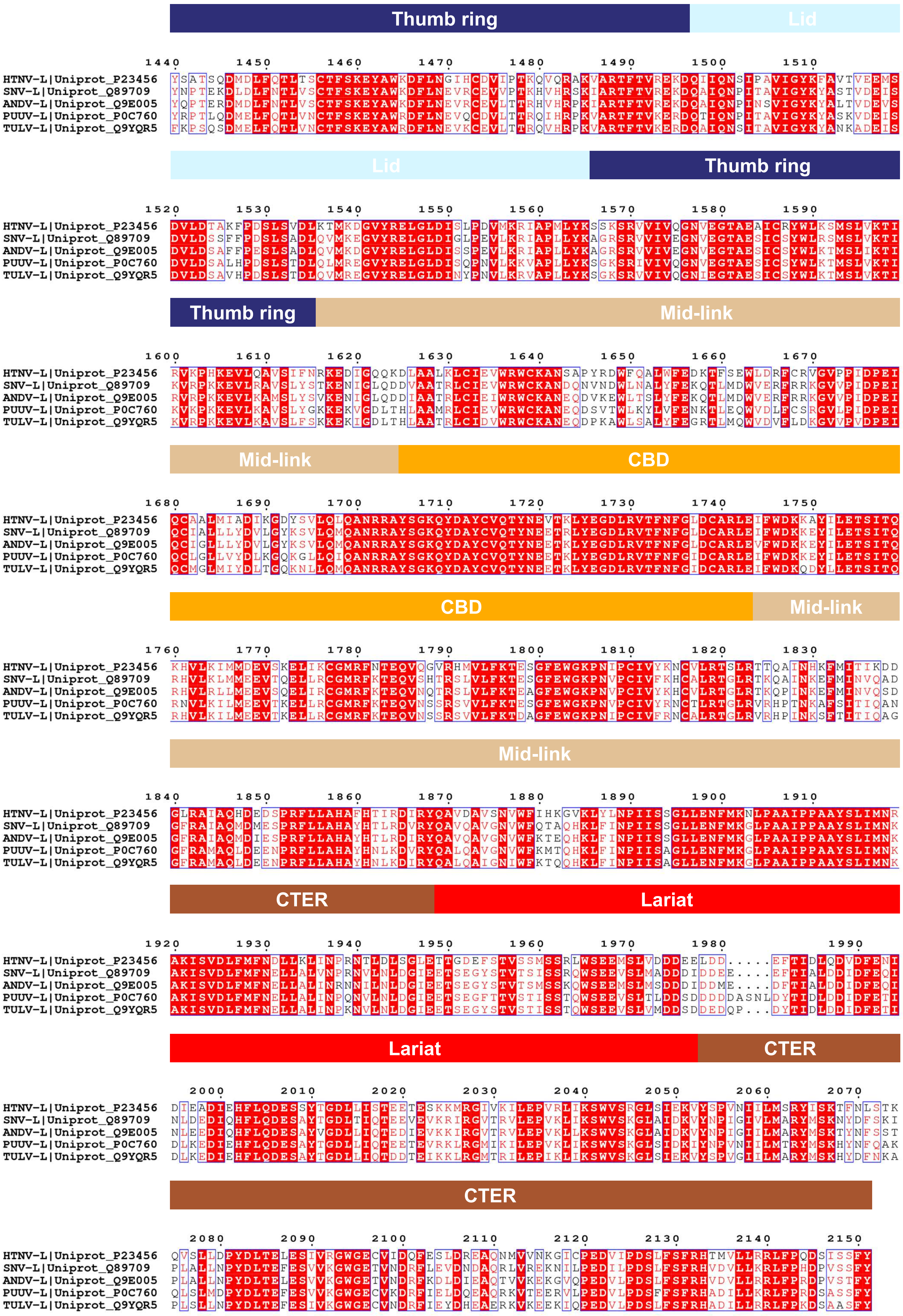

Supplement: Supplementary file 3 — Supplementary Data 1 [file 41467_2024_46601_MOESM3_ESM.pdf]
